# Identification of 20(*S*)-Ginsenoside Rh2 as a Potential EGFR Tyrosine Kinase Inhibitor

**DOI:** 10.1155/2022/6119737

**Published:** 2022-01-24

**Authors:** Yuan Liang, Jingqi Zhao, Haoyang Zou, Jie Zhang, Tiehua Zhang

**Affiliations:** College of Food Science and Engineering, Jilin University, Changchun 130062, China

## Abstract

As the main active ingredients of *Panax ginseng*, ginsenosides possess numerous bioactivities. Epidermal growth factor receptor (EGFR) was widely used as a valid target in anticancer therapy. Herein, the EGFR targeting activities of 20(*S*)-ginsenoside Rh2 (20(*S*)-Rh2) and the relationship of their structure-activity were investigated. Homogeneous time-resolved fluorescence assay showed that 20(*S*)-Rh2 significantly inhibited the activity against EGFR kinase. 20(*S*)-Rh2 was confirmed to effectively inhibited cell proliferation in a dose-dependent manner by MTT assay. Furthermore, quantitative real-time PCR and western blotting analysis revealed that 20(*S*)-Rh2 inhibited A549 cells growth via the EGFR-MAPK pathway. Meanwhile, 20(*S*)-Rh2 could promote cell apoptosis, block cell cycle, and reduce cell migration of A549 cells, respectively. *In silico*, the result suggested that both hydrophobic interactions and hydrogen-bonding interactions could contribute to stabilize their binding. Molecular dynamics simulation showed that the side chain sugar moiety of 20(*S*)-Rh2 was too flexible to be fixed at the active site of EGFR. Collectively, these findings suggested that 20(*S*)-Rh2 might serve as a potential EGFR tyrosine kinase inhibitor.

## 1. Introduction

Lung cancer is the leading cause of cancer death worldwide. Non-small-cell lung cancer (NSCLC) contributes over 80% of lung cancer cases with a low 5-year survival rate [1]. The development of epidermal growth factor receptor-tyrosine kinase inhibitors (EGFR-TKIs) plays a key role in the targeted therapy of NSCLC. Epidermal growth factor receptor (EGFR) is considered a receptor tyrosine kinase with penetrating the cell membrane [2]. EGFR is composed of an extracellular ligand-binding region, a transmembrane region, and an intracellular tyrosine kinase region [3]. Binding to the ligand, EGFR forms a dimer and the phosphate of ATP transfers into the tyrosine residue. Then, different kinds of proteins bind to this phosphorylated tyrosine and signals transmit to downstream pathways, such as mitogen-activated protein kinase (MAPK) and phosphatidylinositol 3-kinase (PI3K) pathways [4, 5]. It is well known that EGFR plays a key role in cell proliferation, apoptosis, and migration [6]. Furthermore, EGFR is confirmed to be dysregulated or overexpressed in various solid tumors and used as one of the valid targets in anticancer therapy [7, 8].

As the main active ingredients of *Panax ginseng* (*P*. *ginseng*), ginsenosides have been widely used in cancer treatment with reduced side effects [9–11]. Ginsenoside Rh2 can be divided into 20(*S*)-ginsenoside Rh2 (20(*S*)-Rh2) and 20(*R*)-ginsenoside Rh2 (20(*R*)-Rh2) according to different orientations of the hydroxyl group at C-20 position [12, 13]. Tumor-associated macrophages (TAMs) are confirmed to play crucial roles in modulating the tumor microenvironment and promoting tumor metastases [14]. Rh2 showed potential to convert TAMs from the alternatively activated M2 macrophages to classically activated M1 macrophages in the microenvironment. Meanwhile, Rh2 prevented NSCLC cell migration, suggesting the therapeutic effects of Rh2 on lung cancer [15]. Rh2 was also demonstrated to inhibit the proliferation and metastasis of NSCLC cells by inducing apoptosis and suppressing epithelial-mesenchymal transition, respectively [16]. In the chemotherapy for NSCLC patients, Rh2 enhanced the antitumor effects of cisplatin through inhibiting the superoxide generation, PD-L1 expression, and cell autophagy [17]. However, the mechanism of 20(*S*)-Rh2 targeting EGFR to inhibit cell proliferation is not clear enough now.

Hence, this work is aimed at identifying 20(*S*)-Rh2 as a potential EGFR tyrosine kinase inhibitor by the combination of *in vitro* and *in silico* approaches. Homogeneous time-resolved fluorescence (HTRF) assay was performed to detect the EGFR kinase activity after the treatment of 20(*S*)-Rh2. Cell viability was measured by MTT assay. To investigate whether 20(*S*)-Rh2 regulated the EGFR-MAPK pathway, the changes in gene expressions and protein contents were determined by quantitative real-time PCR and western blot analysis, respectively. Meanwhile, cell apoptosis and cycle analyses were taken by flow cytometry. Cell wound healing assay was also performed to measure the migration of A549 cells. On this basis, the possible binding conformation of 20(*S*)-Rh2 with EGFR was predicted using molecular docking. The binding stability of the EGFR-20(*S*)-Rh2 complex was explored using molecular dynamics simulation.

## 2. Materials and Methods

### 2.1. Materials

HTRF® KinEASE-TK assay kit was obtained from Cisbio (Codolet, France). EGFR protein (GST-tagged) was obtained from Thermo Fisher Scientific (Carlsbad, CA, USA). Dulbecco's modified Eagle's medium (DMEM, low glucose), penicillin and streptomycin, trypsin, and phosphate buffer saline (PBS) were obtained from Gibco (Paisley, UK). Fetal bovine serum (FBS) was obtained from Zhejiang Tianhang Biotechnology Co., Ltd. (Zhejiang, Hangzhou, China). 3-(4,5-Dimethylthiazol-2-yl)-2,5-diphenyltetrazolium bromide (MTT) was obtained from Shanghai Aladdin Biochemical Technology Co., Ltd. (Shanghai, China). 20(*S*)-Rh2 and bovine serum albumin (BSA) were obtained from Yuanye Biotech Co., Ltd. (Shanghai, China). Dimethyl sulfoxide (DMSO) was obtained from Beijing Solarbio Science & Technology Co., Ltd. (Beijing, China). Ethylenediamine tetraacetic acid (EDTA) was obtained from Sigma-Aldrich (MO, USA). Goat anti-rat IgG secondary antibody was obtained from Sino Biological Inc. (Beijing, China). Antibody against GAPDH was obtained from Gene Tex (Irvine, CA, USA). Other primary antibodies were all obtained from Abcam (Cambridge, MA, USA). All other chemical reagents were of analytical grade.

### 2.2. Measurement of EGFR Kinase Activity by 20(*S*)-Rh2

The enzyme reaction was conducted for 40 min at room temperature in white HTRF 96-well low volume plate. 20(*S*)-Rh2 was 10-fold serial dilutions from 10^3^ to 10^−3^ *μ*M. During the enzymatic step, 20(*S*)-Rh2 (4 *μ*L) and the TK Substrate-biotin (50 *μ*M, 2 *μ*L) were incubated with the EGFR kinase (41 nM, 2 *μ*L); then, ATP (1.41 *μ*M, 2 *μ*L) was added to start the reaction. The enzymatic buffer contained 5 mM MgCl_2_, 1 mM MnCl_2_, and 1 mM DTT. Subsequently, at the detection step, streptavidin-XL665 (16.67 *μ*M, 5 *μ*L) and TK-antibody labeled with Eu^3+^-cryptate (5 *μ*L) in the detection buffer with EDTA were added to stop the kinase activity. After incubation for another 1 h at room temperature, fluorescence was measured at both 620 nm and 665 nm with a microplate reader (Tecan Spark® 10 M, Tecan, Männedorf, Switzerland) [18, 19]. The half maximal inhibitory concentration (IC_50_) was calculated by PRISM version 5.0 software (GraphPad Software Inc., CA, USA).

### 2.3. Cell Culture

The human non-small-cell lung cancer cell line A549 was obtained from Cell Bank of Chinese Academy of Sciences (Shanghai, China). A549 cells were cultured in the DMEM supplemented with 10% FBS, 100 U/mL penicillin, and 0.1 mg/mL streptomycin, at 37°C in a humidified atmosphere of 5% CO_2_.

### 2.4. MTT Assay

A549 cells were seeded in 96-well plates at a density of 1 × 10^4^ cells per well and incubated for 24 h to allow for cell attachment. Then, A549 cells were incubated with different concentrations (5, 10, 20, 30, 40, 50, 60, 80, 100, and 120 *μ*M) of 20(*S*)-Rh2 for another 24 h. The culture medium was removed and replaced with new culture medium with MTT solution (5 mg/mL) and incubated at 37°C for 4 h. Then, the medium was removed and DMSO (110 *μ*L) was added to each well to dissolve the crystals. The absorbance at 490 nm was measured with a microplate reader (Bio-Rad, Hercules, CA, USA). The cell viabilities of A549 cells were calculated as the percentage of absorbance compared to DMSO control cells.

### 2.5. Quantitative Real-Time PCR

A549 cells were seeded in 10 cm discs at a density of 1 × 10^6^ cells per disc and incubated overnight; then, cells were incubated with different concentrations (15, 22, and 35 *μ*M) of 20(*S*)-Rh2 for another 24 h. Total RNA was extracted using TRIzol reagent (Transgen Biotech Co., Ltd., Beijing, China). Reverse transcription and PCR amplification were carried out with TransScript one-step gDNA removal and cDNA synthesis supermix kit (Transgen Biotech Co., Ltd., Beijing, China). Quantitative real-time PCR was conducted using CFX96™ real-time system (Bio-rad®, Hercules, CA, USA) along with the primer pairs listed in the past article of our team [20]. The relative expression levels of mRNA were calculated by the 2^-*ΔΔ*CT^ method normalized with the GAPDH RNA level.

### 2.6. Western Blotting Analysis

A549 cells were seeded in 10 cm discs at a density of 5 × 10^6^ cells per disc and incubated for 24 h; then, cells were incubated with different concentrations (15, 22, and 35 *μ*M) of 20(*S*)-Rh2 for another 24 h. Cell samples were washed with the ice-cold PBS and lysed with RIPA buffer (Cell Signaling Technology, Danvers, MA, USA) containing protease/phosphatase inhibitors (Cell Signaling Technology, Danvers, MA, USA). Cell protein lysates were separated by SDS-polyacrylamide gel electrophoresis (PAGE) and then transferred onto a polyvinylidene difluoride (PVDF) membrane (Millipore, USA). After being blocked with 5% skim milk or BSA dissolved in TBST buffer, the membranes were incubated with primary antibodies at 4°C overnight. Then, goat anti-rat IgG secondary antibody was added. Following the washing step of the TBST buffer, an ECL immunoblotting detection kit (Clinx Science Instrument Co., Ltd., Shanghai, China) was used to detect the visualization. Antibody against GAPDH was used as an internal control.

### 2.7. Analysis of Cell Apoptosis and Cycle by Flow Cytometry

A549 cells were seeded in 6-well plates at a density of 2.5 × 10^5^ cells per well and incubated for 24 h; then, cells were incubated with different concentrations (15, 22, and 35 *μ*M) of 20(*S*)-Rh2 for another 24 h. A549 cells were harvested with EDTA-free trypsin and washed with the ice-cold PBS. Then, cell apoptosis analysis was performed using annexin V-FITC/PI apoptosis detection kit (Meilunbio, Dalian, China). Followed by centrifugation at 1000 g for 5 min, the cells were resuspended in the 1× binding buffer. After annexin V-FITC (5 *μ*L) and PI (7 *μ*L) were added to each group, samples were incubated for 15 min at room temperature. Cell apoptosis analysis was detected with a flow cytometry (Cytoflex, Beckman, Coulter, CA, USA).

On the other hand, cell cycle analysis was performed using PI staining cell cycle detection kit (Meilunbio, Dalian, China). Followed by centrifugation at 1000 g for 5 min, the cells were resuspended in the ice-cold buffer. After fixed with 75% ice-cold ethanol at 4°C overnight, the cells were stained with PI staining buffer at 37°C for 30 min from light. Cell cycle analysis was also detected with a flow cytometry (Cytoflex, Beckman, Coulter, CA, USA).

### 2.8. Wound Healing Assay

Wound healing assay was taken to measure the capacity of 20(*S*)-Rh2 in A549 cells migration and invasion. A549 cells were seeded in 6-well plates at a density of 1 × 10^6^ cells per well; after the cells grew to a monolayer, a pipette tip (200 *μ*L) was used to scraped the monolayer to create an artificial wound field. Then, cells were incubated with different concentrations (15, 22, and 35 *μ*M) of 20(*S*)-Rh2. DMSO was used as a blank control. Photographs were taken at 0, 6, 12, and 24 h using an inverted microscope (Olympus, Tokyo, Japan). The surface areas of cell wound were quantified by ImageJ software (National Institute of Health, Bethesda, MD, USA).

### 2.9. Molecular Docking

Based on the structure of EGFR-erlotinib complex (PDB 1 M17), molecular docking was performed to predict the possible binding conformation of 20(*S*)-Rh2 with EGFR [21]. The cocrystallized ligand erlotinib as well as all water molecules were dislodged using Chimera 1.11 to complete the preparation of EGFR structure. The hydrogen atoms were added using AutoDockTools-1.5.6 [22]. The structure of 20(*S*)-Rh2 was generated using GaussView 5.0 and then was subject to the energy minimization using Gaussian 09 W [23]. Afterward, 20(*S*)-Rh2 was docked to the binding pocket of EGFR with a Lamarckian genetic algorithm using AutoDockTools-1.5.6. The grid box was generated at the center of the cocrystallized ligand erlotinib. Taken together with the above docking parameters, 10 independent calculations were performed and the conformation of 20(*S*)-Rh2 with lowest binding energy was visualized using PyMOL.

### 2.10. Molecular Dynamics Simulation

Based on the optimal conformation of 20(*S*)-Rh2 obtained from molecular docking, a 20 ns molecular dynamics simulation was performed using GROMACS 2019 [24]. The topologies of EGFR and 20(*S*)-Rh2 were developed using CHARMM36 all-atom force field and CGenFF server, respectively [25, 26]. Afterward, the EGFR-20(*S*)-Rh2 complex was placed in a cubic box filling with water. The whole system was appended with counterion to obtain electrostatic neutrality. Energy minimization was completed followed with constant NVT (number of particles-volume-temperature) and constant NPT (number of particles-pressure-temperature) equilibration procedure for 0.1 ns. With the desired temperature and pressure, the position restraint on the system was released. On this basis, molecular dynamics simulation was run and the data was collected to calculate the root mean squared deviation (RMSD) values.

### 2.11. Statistical Analysis

All experiments were performed independently at least three times, and all the data were expressed as mean ± standard error of measurement (SEM). Analysis of variance (ANOVA) was used to assess the statistical significance of experimental manipulations. The difference compared with the control group was considered statistically significant at ^∗^*p* < 0.05, ^∗∗^*p* < 0.01, and ^∗∗∗^*p* < 0.001.

## 3. Results and Discussion

### 3.1. 20(S)-Rh2 Inhibited the Activity of EGFR Kinase

**Figure 1:**
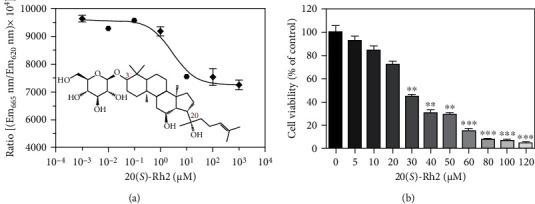
(a) 20(*S*)-Rh2 inhibited the activity of EGFR kinase and (b) A549 cell proliferation. DMSO-treated group was used as control. ^∗^*p* < 0.05, ^∗∗^*p* < 0.01, and ^∗∗∗^*p* < 0.001.

HTRF assay is a homogeneous time-resolved assay that generates a signal by fluorescence resonance energy transfer (FRET) between the donor and acceptor molecules [27]. The donor is a Eu^3+^ caged in the polycyclic cryptate, while the acceptor is a streptavidin-XL665 labeled protein. Laser excitation of the donor at 337 nm leads to the transfer of energy to the acceptor at 620 nm when they are in the close proximity, resulting in the emission of light over a prolonged period of milliseconds at 665 nm [28]. In this assay, HTRF was performed to analyze the effect of 20(*S*)-Rh2 on EGFR kinase activity. 20(*S*)-Rh2 altered EGFR kinase activity in a dose-dependent manner, and the inhibitory curve was presented in Figure 1(a). The IC_50_ of 20(*S*)-Rh2 on EGFR kinase activity was determined to be 2.78 ± 0.16 *μ*M with the average of at least three independent experiments. The data demonstrated that 20(*S*)-Rh2 inhibited the activity of EGFR kinase.

### 3.2. Effect of 20(S)-Rh2 on the Inhibition of A549 Cell Proliferation

Exponentially growing A549 cells were seeded continuously in the presence or absence of different concentrations of 20(*S*)-Rh2. The effect of 20(*S*)-Rh2 on cell growth was assessed by the MTT assay. As shown in Figure 1(b), 20(*S*)-Rh2 inhibited the growth of A549 cells in a dose-dependent manner. DMSO was used as a blank control. The 20%, 40%, and 60% inhibitory concentrations (IC_20_, IC_40_, and IC_60_) of 20(*S*)-Rh2 on A549 cells were 15, 22, and 35 *μ*M, respectively.

### 3.3. 20(S)-Rh2 Regulated the EGFR-MAPK Pathway in A549 Cells

**Figure 2:**
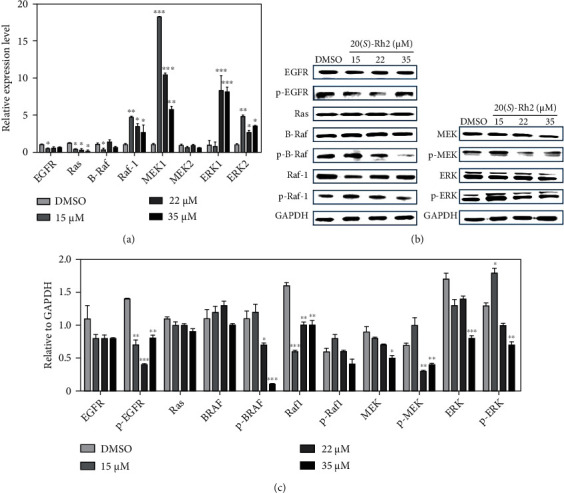
20(*S*)-Rh2 regulated the EGFR-MAPK pathway in A549 cells. (a) Quantitative real-time PCR and (b) western blot analysis of 20(*S*)-Rh2 to EGFR-MAPK pathway. (c) Quantitative analyses of the levels of EGFR-MAPK pathway related proteins compared to GAPDH. ^∗^*p* < 0.05, ^∗∗^*p* < 0.01, and ^∗∗∗^*p* < 0.001.

To investigate whether 20(*S*)-Rh2 regulated the MAPK pathway through affecting the expressions of EGFR, the changes in gene expressions and protein contents were determined by quantitative real-time PCR and western blot analysis, respectively. As shown in Figure 2(a), after the treatment of 20(*S*)-Rh2, the gene expression of *EGFR* was all downregulated. Similarly, the protein contents of total-EGFR and phospho-EGFR were all downregulated after the treatment of 20(*S*)-Rh2 compared to the DMSO-treated group (Figures 2(b) and 2(c)). Combined with the result of HTRF, 20(*S*)-Rh2 could inhibit the phosphorylation of EGFR. After the treatment of 20(*S*)-Rh2, the gene expressions of *Ras* and *BRAF* were downregulated (Figure 2(a)); the protein content of Ras was also slightly downregulated; however, the protein content of BRAF was firstly upregulated then downregulated. Especially, the protein content of phospho-BRAF was significantly downregulated at the concentrations of 35 *μ*M (Figure 2(c)). The gene expression of *Raf1* was upregulated after the treatment of 20(*S*)-Rh2; however, the protein content of Raf1 was significantly downregulated (Figure 2(c)). Apart from the gene expression of *MEK2* downregulated, the gene expressions of *MEK1*, *ERK1*, and *ERK2* were all significantly upregulated after the treatment of 20(*S*)-Rh2 (Figure 2(a)). As for the protein contents, phospho-MEK and phospho-ERK were upregulated at 15 *μ*M and then downregulated at 22 and 35 *μ*M (Figure 2(c)); total-MEK and total-ERK were downregulated (Figure 2(c)). Taken together, the results indicated that 20(*S*)-Rh2 inhibited the activation of EGFR to regulate the MAPK pathway leading to suppress A549 cells growth.

### 3.4. 20(S)-Rh2 Promoted the Apoptosis of A549 Cells

**Figure 3:**
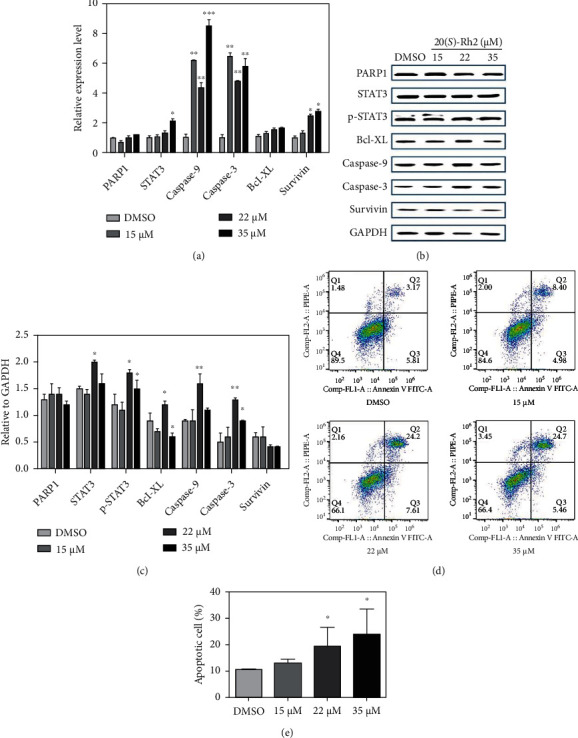
20(*S*)-Rh2 promoted the apoptosis of A549 cells. The effect of 20(*S*)-Rh2 on A549 cells apoptosis was evaluated by (a) quantitative real-time PCR and (b) western blot analysis. (c) Quantitative analyses of the levels of cell apoptosis-related proteins compared to GAPDH. The results of (d) flow cytometry and (e) apoptotic cells of each group. ^∗^*p* < 0.05, ^∗∗^*p* < 0.01, and ^∗∗∗^*p* < 0.001.

PARP1 is the first member of the PARP family that acts as a DNA damage sensor [29]. After binding on DNA damaged structures, PARP1 serves as a survival factor and recruits repair enzymes [30]. Caspases cleave various proteins that are necessary for the cell survival and function during the apoptosis phase [31]. PARP1 protein is cleaved by caspase-3 and caspase-7 to retain basal enzymatic activity without to be stimulated by DNA damage [32, 33]. Through this process, the cleavage of PARP1 may help to induce cells to the apoptotic pathway. As shown in Figure 3(a), after the treatment of 20(*S*)-Rh2, although the gene expression of *PARP1* was nearly unchanged, the protein content of PARP1 was firstly upregulated then downregulated at 35 *μ*M (Figures 3(b) and 3(c)) to induce A549 cell apoptosis.

The caspase cascade system plays a crucial role in the induction, transduction, and amplification of cell intracellular apoptotic signals [34, 35]. Once caspases activated, the cells are dismantled by selectively cleaving key proteins. Caspase-9 is activated after cytochrome c (Cyt c) was released; then, effector caspases and Bid are activated to remodel the mitochondria [36]. However, the process of cell death is promoted by caspase-3, which is the primary executioner of apoptosis. In this work, as for the caspase-9 and caspase-3, their gene expressions were significantly upregulated after the treatment of 20(*S*)-Rh2; meanwhile, their protein contents were also upregulated, especially at 22 *μ*M (Figure 3(c)). As a member of the inhibitor of apoptosis family, survivin expressed in various human cancers may induce evasion from aberrant mitotic and apoptosis progression. The treatment of 20(*S*)-Rh2 downregulated the protein content of survivin at 22 and 35 *μ*M (Figure 3(c)) to induce A549 cell apoptosis.

Annexin V-FITC/PI staining was performed on the A549 cells, and the fluorescence was recorded using flow cytometry (Supplementary Figure S1). After the treatment of 20(*S*)-Rh2, the number of necrotic cells at Q1 and early apoptotic cells at Q3 changed little. However, the number of late apoptotic cells at Q2 significantly increased, especially at 22 and 35 *μ*M (Figure 3(d)). The total apoptotic cells also increased in a dose-dependent manner (Figure 3(e)). Taken the results together, 20(*S*)-Rh2 could promote the apoptosis of A549 cells.

### 3.5. 20(S)-Rh2 Blocked A549 Cell Cycle at G_0_/G_1_ Phase

**Figure 4:**
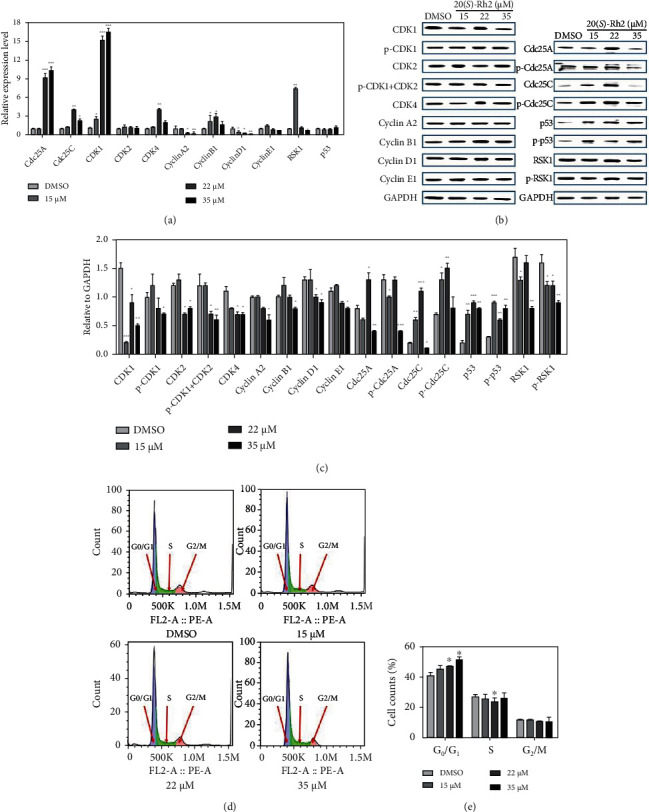
20(*S*)-Rh2 blocked A549 cells cycle at G_0_/G_1_ phase. The effect of 20(*S*)-Rh2 on A549 cells cycle was evaluated by (a) quantitative real-time PCR and (b) western blot analysis. (c) Quantitative analyses of the levels of cell cycle-related proteins compared to GAPDH. The results of (d) flow cytometry and (e) cell counts of each group. ^∗^*p* < 0.05, ^∗∗^*p* < 0.01, and ^∗∗∗^*p* < 0.001.

As key regulators of the cell cycle, cell division cycle 25 (Cdc25) phosphatase family mainly contain Cdc25A, Cdc25B, and Cdc25C [37]. Although sharing the common biochemical mechanism of action, members of Cdc25 phosphatase family have unique characteristics and play specific roles in the cell cycle regulation [38]. Cdc25A plays a crucial role at the transition of G_1_ to S phase [39]. Cdc25B is activated during S phase and induces the activation of cyclin-dependent protein kinase 1- (CDK1-) cyclin B in the cytoplasm [40]. Then, activated CDK1-cyclin B phosphorylates and activates Cdc25C resulting in a positive feedback mechanism and entering into mitosis [41]. As shown in Figure 4(a), after the treatment of 20(*S*)-Rh2, although the gene expression of *Cdc25A* was upregulated at high concentrations, the protein contents of total-Cdc25A and phospho-Cdc25A were initially upregulated and then downregulated at 35 *μ*M (Figures 4(b) and 4(c)). Thus, high concentrations of 20(*S*)-Rh2 could downregulate the expression of Cdc25A to inhibit the transition of G_1_ to S phase.

The phosphorylation of CDKs promotes the process of cell cycle. CDKs are initially activated by the combination of cyclin subunits, as well as the phosphorylation on the threonine residue in a conserved amino-acid sequence [42]. After cyclin E complexed with CDK2, this complex entries cell cycle into S phase and induces the initiation of DNA replication [43]. Subsequently, cyclin A is expressed at the boundary of G_1_ to S phase and forms complex with CDK2 [44]. Then, the complex of cyclin A-CDK2 is necessary for both S phase transition and DNA replication [45]. It is determined that after the treatment of 20(*S*)-Rh2, the gene expressions of *cyclin A*, *cyclin E*, and *CDK2* were all downregulated, especially for *cyclin A* (Figure 4(a)). Furthermore, the protein contents of cyclin A, cyclin E, and CDK2 were initially upregulated and then downregulated at high concentrations (Figure 4(c)). The downregulated contents of cyclin A, cyclin E, and CDK2 inhibited the formation of cyclin A-CDK2 and cyclin E-CDK2 to block cell cycle at G_0_/G_1_ phase.

As a transcription factor, p53 is highly inducible by various stress signals, such as oncogene activation, DNA damage, and nutrient deprivation [46]. Activation of the p53 can lead to cell cycle arrest and cell apoptosis [47]. The crucial mechanism of p53-mediated arrest is the transcriptional downregulation of many cell cycle-related genes [41]. As shown in Figure 4(c), after the treatment of 20(*S*)-Rh2, the protein contents of total-p53 and phospho-p53 were all significantly upregulated to arrest cell cycle.

PI staining was performed on the A549 cells, and the fluorescence was recorded using flow cytometry (Supplementary Figure S2). After the treatment of 20(*S*)-Rh2, the number of cell counts at G_0_/G_1_ phase was increased in a dose-dependent manner. Meanwhile, the number of cell counts at S or G_2_/M phase was decreased compared to the DMSO control groups (Figures 4(d) and 4(e)). Taken the results together, 20(*S*)-Rh2 could block A549 cell cycle at G_0_/G_1_ phase.

### 3.6. 20(S)-Rh2 Reduced A549 Cell Migration

**Figure 5:**
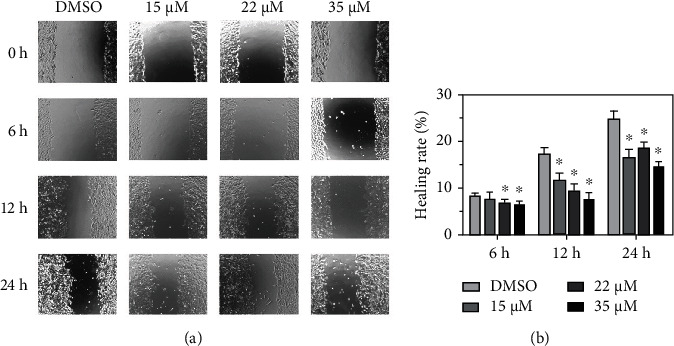
20(*S*)-Rh2 reduced A549 cell migration. (a) The wound healing assay was performed on A549 cells after the treatment of 20(*S*)-Rh2. (b) Healing rate of each group. DMSO was used as control. ^∗^*p* < 0.05.

The inhibition activity of 20(*S*)-Rh2 on A549 cell migration was assessed by cell wound healing assay [48]. Photomicrographs were taken at 0, 6, 12, and 24 h after wounding (Figure 5(a)). The DMSO-treated group was used as a control. As a percentage of the initial wound area, the remaining cell-free area was taken as an index of wound healing [49, 50]. In the DMSO control group, about 8% of the wound area healed at 6 h after wounding compared to the 0 h. Meanwhile, the wound area of the 20(*S*)-Rh2-treated group significantly healed at 22 *μ*M (6.8%) and 35 *μ*M (6.4%). After the treatment of 20(*S*)-Rh2 at 12 h, the migration rate of the transfected A549 cells significantly reduced in a dose-dependent manner. The healing rate reduced to 7.5% at 35 *μ*M of 20(*S*)-Rh2 compared to the 17.3% of the DMSO-treated group. Interestingly, the healing rate also significantly reduced at 24 h indicated that 20(*S*)-Rh2 inhibited the A549 cell mobility at 24 h. In general, after the treatment of 20(*S*)-Rh2, the closure of the wounded area healed slowly (Figure 5(b)). Therefore, 20(*S*)-Rh2 could reduce A549 cell migration.

### 3.7. Possible Binding Conformation of 20(S)-Rh2 with EGFR

**Figure 6:**
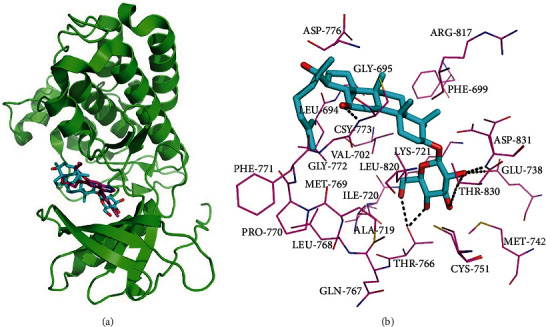
The result of molecular docking. (a) Docked 20(*S*)-Rh2 (cyan sticks) and cocrystallized ligand erlotinib (magenta sticks) in the binding pocket of EGFR (green cartoon). (b) Amino acid residues (magenta lines) of EGFR that lie within 4 Å away from 20(*S*)-Rh2 (cyan sticks). The hydrogen bonds were shown as black dotted lines.

The result of molecular docking suggested that 20(*S*)-Rh2 was accommodated in the binding pocket of EGFR, adopting a similar binding conformation with that of co-crystallized ligand erlotinib (Figure 6(a)). As shown in Figure 6(b), 20(*S*)-Rh2 made hydrophobic interactions with 11 amino acid residues, including Leu694, Phe699, Val702, Ala719, Ile720, Met742, Leu768, Met769, Pro770, Phe771, and Leu820. The side chain sugar moiety attached at C-3 position (Figure 1(a)) formed hydrogen bonds with residues Glu738, Thr766, Thr830, and Asp831. Meanwhile, the hydroxyl group at primary ring also formed a hydrogen bond with residue Cys773. Collectively, these findings suggested that both hydrophobic interactions and hydrogen-bonding interactions could contribute to stabilize the EGFR-20(*S*)-Rh2 binding.

### 3.8. Binding Stability of the EGFR-20(S)-Rh2 Complex

**Figure 7:**
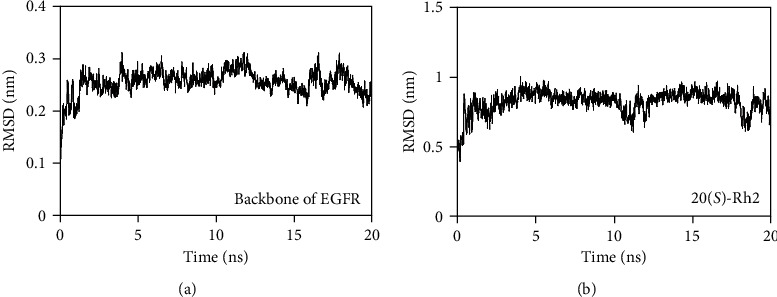
Variations of root mean square deviation (RMSD) values for the (a) backbone of EGFR and (b) 20(*S*)-Rh2 during a 20 ns molecular dynamics simulation.

In this work, a 20 ns molecular dynamics simulation was performed to explore the binding stability of the EGFR-20(*S*)-Rh2 complex. The backbone of EGFR basically kept stable during the whole simulation process with the average RMSD values of 0.25 ± 0.03 nm (Figure 7(a)), indicating that 20(*S*)-Rh2 caused little conformational change toward EGFR. Conversely, 20(*S*)-Rh2 underwent a relatively severe disturbance during 20 ns molecular dynamics simulation, with the average RMSD values of 0.82 ± 0.08 nm (Figure 7(b)). As shown in Figures 1(a) and 6(a), the side chain sugar moiety of 20(*S*)-Rh2 was too flexible to be fixed at the active site of EGFR, which might be responsible for the structural disturbance of 20(*S*)-Rh2.

## 4. Conclusion

HTRF assay was taken to confirm that 20(*S*)-Rh2 significantly inhibited the activity of EGFR kinase. The changes in gene expressions and protein contents demonstrated that 20(*S*)-Rh2 regulated the EGFR-MAPK pathway to inhibit A549 cell proliferation. The findings of the present study suggested that 20(*S*)-Rh2 could promote cell apoptosis, block cell cycle, and reduce cell migration of A549 cells. The result of molecular docking suggested that both hydrophobic interactions and hydrogen-bonding interactions could contribute to stabilize the EGFR-20(*S*)-Rh2 binding. Further exploration of their binding stability was also performed by molecular dynamics simulation. Collectively, these findings suggested that 20(*S*)-Rh2 might serve as a potential EGFR tyrosine kinase inhibitor.

## Data Availability

The data used to support the findings of this study are available from the corresponding author upon request.

## Supplementary Materials

Supplementary Materials Supplementary Figure S1: related to Figure 3. Cell apoptosis assay by flow cytometry. Supplementary Figure S2: related to Figure 4. Cell cycle assay by flow cytometry.
