# Supplementary figures and images for: Identification of 20(S)-Ginsenoside Rh2 as a Potential EGFR Tyrosine Kinase Inhibitor
Source: Oxid Med Cell Longev. 2022 Jan 24;2022:6119737. doi: 10.1155/2022/6119737 (PMC8803441; doi:10.1155/2022/6119737)

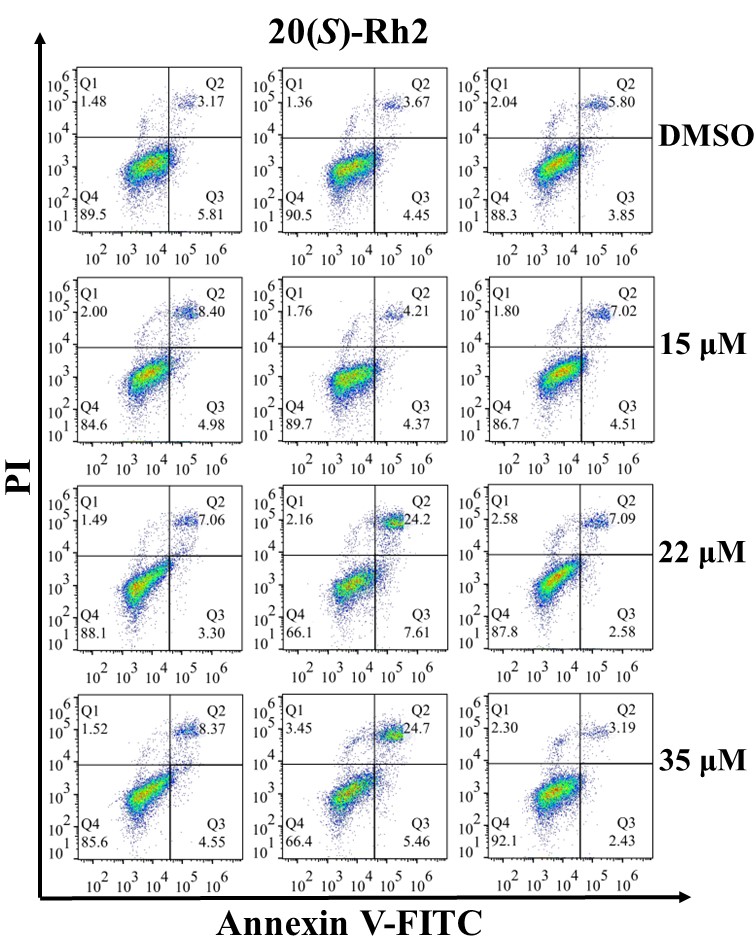
 Figure S1: Cell apoptosis assay by flow cytometry.


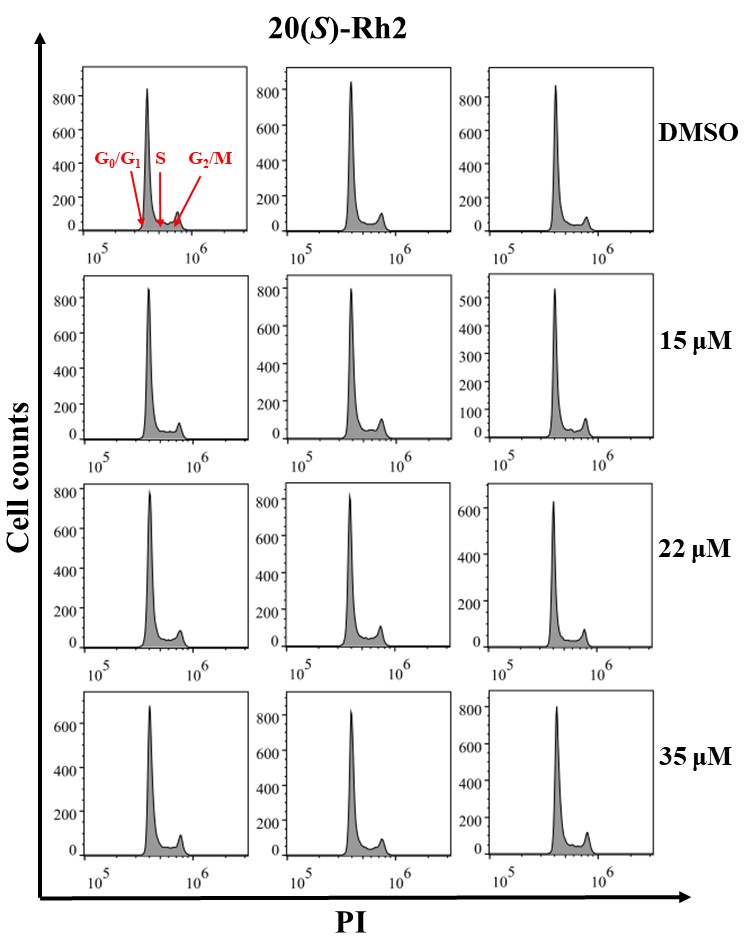
 Figure S2: Cell cycle assay by flow cytometry.

Supplement: Supplementary Materials — Supplementary Figure S1: related to Figure 3. Cell apoptosis assay by flow cytometry. Supplementary Figure S2: related to Figure 4. Cell cycle assay by flow cytometry. [file 6119737.f1.docx]
